# Patchoulene Epoxide Isolated from Patchouli Oil Suppresses Acute Inflammation through Inhibition of NF-*κ*B and Downregulation of COX-2/iNOS

**DOI:** 10.1155/2017/1089028

**Published:** 2017-07-25

**Authors:** Jia-Li Liang, Jia-Zhen Wu, Yu-Hong Liu, Zhen-Biao Zhang, Qi-Duan Wu, Han-Bin Chen, Yan-Feng Huang, Yao-Xing Dou, Jiang-Tao Zhou, Zi-Ren Su, Janis Ya-Xian Zhan

**Affiliations:** ^1^Mathematical Engineering Academy of Chinese Medicine, Guangzhou University of Chinese Medicine, Guangzhou 510006, China; ^2^Guangdong Provincial Key Laboratory of New Drug Development and Research of Chinese Medicine, Guangzhou University of Chinese Medicine, Guangzhou 510006, China; ^3^The First Affiliated Hospital of Chinese Medicine, Guangzhou University of Chinese Medicine, Guangzhou 510405, China; ^4^Dongguan Mathematical Engineering Academy of Chinese Medicine, Guangzhou University of Chinese Medicine, Dongguan 523808, China

## Abstract

According to the GC-MS analysis, compositional variation was observed between samples of patchouli oil, of which an unknown compound identified as patchoulene epoxide (PAO) was found only in the long-stored oil, whose biological activity still remains unknown. Therefore, the present study aimed to evaluate the potential anti-inflammatory activity with three in vivo inflammatory models: xylene-induced ear edema, acetic acid-induced vascular permeability, and carrageenan-induced paw edema. Further investigation into its underlying mechanism on carrageenan-induced paw edema was conducted. Results demonstrated that PAO significantly inhibited the ear edema induced by xylene, lowered vascular permeability induced by acetic acid and decreased the paw edema induced by carrageenan. Moreover, PAO markedly decreased levels of tumor necrosis factor-*α* (TNF-*α*), interleukin-1*β* (IL-1*β*), interleukin-6 (IL-6), prostaglandin E2 (PGE_2_), and nitric oxide (NO), but increased levels of interleukin-4 (IL-4) and interleukin-10 (IL-10). PAO was also shown to significantly downregulate the protein and mRNA expressions of cyclooxygenase-2 (COX-2) and inducible nitric-oxide synthase (iNOS). Western blot analysis revealed that PAO remarkably inhibited p50 and p65 translocation from the cytosol to the nucleus by suppressing IKK*β* and I*κ*B*α* phosphorylation. In conclusion, PAO exhibited potent anti-inflammatory activity probably by suppressing the activation of iNOS, COX-2 and NF-*κ*B signaling pathways.

## 1. Introduction

Acute inflammation is an initial protective response of an immunological defense system to harmful stimuli such as microbial infections, allergens, and physical injuries [1], which is characterized by redness, swelling, heat, and arteriosclerosis [2]. Pathogenesis of acute inflammation involves various signaling molecules such as COX-2, iNOS, and NF-*κ*B [3]. Growing evidence demonstrates that acute inflammation plays an important role in maintenance of the organic integrity and is related to various diseases such as diabetes, cardiovascular dysfunction, and cancer [4]. All the time, nonsteroidal anti-inflammatory drugs (NSAIDs) are the most commonly prescribed therapeutics for inflammatory diseases. However, they become increasingly controversial due to their side effects like gastric lesions [5]. Therefore, in recent decades, more and more attention has been paid to seeking alternatives with fewer side effects, and a number of natural compounds derived from traditional Chinese herbs such as curcumin and quercetin [6] have been found to have great anti-inflammatory potential.

*Pogostemon cablin* (Blanco) Benth. (Labiatae) is an aromatic herb native to the Philippines and has been widely cultivated in Southeast Asia [7]. As a traditional Chinese medicinal plant, *P. cablin* is commonly used to resolve dampness, dispel summerheat, and relieve fatigue. Patchouli oil is the major active component of *P. cablin*, which is also highly valuable in perfumery and aromatherapy [8]. According to Najafian [9] and Santana et al. [10], storage conditions such as storage time exert great influence on the quality of essential oil. Therefore, in the present work, samples of patchouli oil with different storage time were assayed by GC-MS for the identification of potential compositional variation. Interestingly, a new compound was found and isolated from the patchouli oil with ten-year storage time. This unknown compound was identified as patchoulene epoxide (PAO), which was confirmed by 1D NMR, 2D NMR, and mass spectrometry (MS). Considering that the chemical structure of PAO is highly similar to that of *β*-patchoulene (PAE), which has been reported to exert an anti-inflammatory effect [11], it is reasonable to speculate that PAO may possess an anti-inflammatory effect.

In this study, we conducted an investigation to demonstrate whether PAO could suppress inflammation. A xylene-induced ear edema test, acetic acid-induced peritoneal vascular permeability test, and carrageenan-induced paw edema test were carried out to verify the anti-inflammatory activity of PAO in vivo. To further clarify its anti-inflammatory mechanism, inflammatory mediators including tumor necrosis factor-*α* (TNF-*α*), interleukin-1*β* (IL-1*β*), interleukin-6 (IL-6), interleukin-10 (IL-10), interleukin-4 (IL-4), prostaglandin E2 (PGE_2_), and nitric oxide (NO) were measured in the carrageenan-induced paw edema mouse model. Furthermore, the COX-2, iNOS, and NF-*κ*B signaling pathways were also assayed.

## 2. Materials and Methods

### 2.1. Chemicals and Reagents

Indomethacin (Indo) was purchased from Huanan Pharmacy Company (Guangzhou, China). Evans blue and carrageenan were purchased from Sigma-Aldrich (St. Louis, MO, USA). Tween-80 was purchased from Sinopharm (Shanghai, China). Enzyme-linked immunosorbent assay (ELISA) kits were purchased from eBioscience (San Diego, CA, USA). Nitric oxide (NO) and total protein kits were purchased from Nanjing Jiancheng Bioengineering Institute (Nanjing, Jiangsu, China). All other chemicals were of analytical grade unless stated specifically.

### 2.2. Plant Material and Preparation

Patchouli oil was obtained from Guangzhou Baihua Flavours and Fragrances Company Ltd. (Guangzhou, China). Fresh patchouli oil with one-year storage time (Lot. 150305) and stale patchouli oil with ten-year storage time (Lot. 060713) were analyzed by gas chromatography-mass spectrometry (GC-MS), on an Agilent GC 7890A/MSD5975C instrument (Agilent Technologies Co. Ltd., Santa Clara, CA, USA). Samples (2 *μ*l) dissolved in diethyl ether were injected onto the HP-5MS column (30 m × 250 *μ*m × 0.5 *μ*m). The temperature program was started at 100°C and maintained for 1 min, followed by a gradient increase to 240°C at a rate of 8°C/min and kept for 5 min. The inlet temperature was 250°C. The helium carrier gas was applied to maintain the pressure of 12 psi on the column with a constant flow rate of 1.3 ml/min. Ion source temperature for mass spectrometer was 230°C, operated in a splitless EI mode at 70 eV. The mass spectrum plot was demanded using a full-scan monitoring mode with a mass scan range of *m*/*z* 35–400, and the splitting ratio was 60 : 1. The resulting peaks were identified by comparing the mass spectrums and retention index of compounds to those in the databases and values reported in the literatures.

By comparing the GC-MS spectrums of fresh and stale patchouli oils, an unknown compound was identified which only existed in the stale patchouli oil. Considering that no data were available for this unknown compound, further investigation into its chemical structure was necessary. In this study, components of stale patchouli oil were separated using fractional distillation according to their different volatilities. Stale patchouli oil (1000 g) was placed in the two-neck round-bottom flask of fractionating tower followed by distillation under the vacuum of 6 kPa. The stale patchouli oil was heated at 180°C until refluxing and being maintained for 2 h. Afterwards, with temperature increasing per 2°C, the distillate was collected and analyzed by GC-MS. It was observed that most of the unknown compounds existed in the distillation at 191°C. Subsequently, the distillation at 191°C was chromatographed over silica gel by eluting with petroleum ether. Fractions were collected and then subjected to evaporation under vacuum. The purity of the unknown compound was monitored by GC-MS. The identification was confirmed by 1D NMR, 2D NMR, and MS. Its relative configuration was confirmed by NOESY.

### 2.3. Animals

Kunming (KM) mice of either sex, weighting 18–22 g, were provided by the Laboratory Animal Centre, Guangzhou University of Chinese Medicine (number 44005900002454). The experimental protocol was duly approved according to the NIH Guidelines for the Care and Use of Laboratory Animals. Animals were acclimatized to a constant environment (temperature 22 ± 2°C, humidity 50 ± 10%, and 12 h dark-light cycle) for one week. Food and water were available ad libitum.

### 2.4. Acute Toxicity Test

An acute toxicity test of PAO was performed in mice according to the Food Security of the PRC National Standard (GB 15193.3–2014). Briefly, different doses of PAO (0, 100, 316, 1000, 3160, and 10000 mg/kg) were dissolved in 0.5% Tween-80 solution and given to KM mice (10 mice per group of either sex) by a single oral administration, respectively. After administration, all mice were observed consecutively for two weeks under the normal circumstances with forage and water provided ad libitum. Mortality and clinical toxicity indications were recorded daily.

### 2.5. Xylene-Induced Ear Edema Test

Animals of either sex were randomly assigned to five groups (*n* = 10) for intragastric administration: vehicle (0.5% Tween-80), Indo (indomethacin, 10 mg/kg, positive drug), PAO (10, 20, and 40 mg/kg for PAO-L, PAO-M, PAO-H, resp.). The dosages of Indo and PAO were selected according to Zhang et al. [11] and our preliminary experiment. All groups received prophylactic administration for 7 consecutive days. The xylene-induced ear edema test was carried out as described previously with some modifications [12]. Briefly, after 60 min of the last administration, 20 *μ*l xylene was applied on both surfaces of the right ear to induce ear edema and the left ear served as a self-control. All mice were sacrificed after xylene application for 1 h. Both ears were removed and tailored into circular sections with an 8 mm diameter punch. The ear edema rate (ER) was calculated with the equation as follows:

(1) $$\begin{array}{c} ER\;\left(\%\right)=\frac{W_{r}-W_{l}}{W_{l}}\times 100, \end{array}$$

where *W*_r_ is the weight of the right ear and *W*_l_ is the weight of the left ear.

The inhibition (%) of ear edema was calculated as follows:

(2) $$\begin{array}{c} inhibition\;\left(\%\right)=\frac{E_{v}-E_{t}}{E_{t}}\times 100, \end{array}$$

where *E*_t_ is the average edema rate of the treatment group while *E*_v_ is the average edema rate of the vehicle group.

### 2.6. Acetic Acid-Induced Peritoneal Vascular Permeability Test

Mice were administrated in the same way as that in the xylene-induced ear edema test. The acetic acid-induced peritoneal vascular permeability test was carried out according to Zhang et al. with some modifications [13]. Briefly, 60 min after the final administration, mice were injected intravenously with 1% Evans blue solution (10 ml/kg) followed by an intraperitoneal injection of 0.6% acetic acid (0.1 ml/10 g). Twenty minutes after acetic acid injection, mice were sacrificed and the pigment that leaked into the abdominal cavity was rinsed two times with a total of 5 ml physiological saline solution. After that, the washing solution was centrifuged at 550 ×g for 15 min and the absorbance of the supernatant was measured at 590 nm with a Thermo Scientific Microplate Reader. To evaluate the inhibitory effect of PAO on the enhanced vascular permeability induced by xylene, the following equation was used:

(3) $$\begin{array}{c} inhibition\;\left(\%\right)=\frac{E_{v}-E_{t}}{E_{v}}\times 100, \end{array}$$

where *E*_t_ is the average absorbance value of the treatment groups while *E*_v_ is the average absorbance value of the vehicle group.

### 2.7. Carrageenan-Induced Paw Edema Test

Grouping and administration were performed the same as above. The carrageenan-induced paw edema test was employed according to Deng et al. [14] with some modifications. Briefly, 60 min after the final administration, paw volume was measured as the basal volume (*V*_o_) prior to intraplantar injection of 1% carrageenan suspended in saline (0.05 ml/20 g) into the right hind paws. At different time intervals (1, 2, 3, 4, 5, and 6 h) after carrageenan injection, the paw volume was also measured as the pathological volume (*V*_i_). A MK101CMP plethysmometer (Muromachi Kikai Co. Ltd., Japan) served as the volume-measuring instrument. The paw edema rate (PER) was calculated with the equation as follows:

(4) $$\begin{array}{c} PER\;\left(\%\right)=\frac{V_{i}-V_{o}}{V_{o}}\times 100. \end{array}$$

The inhibition (%) of paw edema was calculated as follows:

(5) $$\begin{array}{c} inhibition\;\left(\%\right)=\frac{E_{v}-E_{t}}{E_{v}}\times 100, \end{array}$$

where *E*_t_ is the average paw edema rate of the treatment groups and *E*_v_ is the average paw edema rate of the vehicle group.

In the following experiment, animals of either sex were randomly divided into six groups (*n* = 18) for intragastric administration: intact and vehicle (0.5% Tween-80), Indo (indomethacin, 10 mg/kg, positive drug), and PAO (10, 20, and 40 mg/kg for PAO-L, PAO-M, and PAO-H, resp.). Sixty minutes after the final administration, all mice except those of the intact group received carrageenan treatment. Four hours after carrageenan injection, mice were sacrificed and right hind paws were dissected immediately for a further inflammatory factor mediator assay.

#### 2.7.1. Cytokine Determination by ELISA

The paws were homogenized in ice-cold PBS (1 : 9, *v*/*w*) to give a 10% homogenate suspension. The supernatants were assayed for TNF-*α*, IL-1*β*, IL-6, IL-10, IL-4, and PGE_2_ levels in accordance with the manufacturer's instructions using commercially available ELISA kits. The absorbances of TNF-*α*, IL-1*β*, IL-6, IL-10, IL-4, and PGE_2_ were determined at 450 nm from standard curves. The results were expressed as pg/mg protein.

#### 2.7.2. NO Assay

NO production was indirectly assessed by measuring the nitrite concentration according to the manufacturer's guidelines. Briefly, the paws were homogenized in ice-cold saline (1 : 9, *v*/*w*) to give a 10% homogenate suspension. The supernatants were applied to 96-well plates and mixed with the Griess reagent. After incubation for 10 min at room temperature, the absorbance was measured at 550 nm. NaNO_2_ was used to generate a standard curve. The content of nitrite oxide was expressed as *μ*mol/g protein.

#### 2.7.3. Quantification of iNOS and COX-2 mRNA Expression in Mouse Paw Tissue via RT-PCR

Total RNA was isolated from paw tissue homogenates using TRIzol® Reagent (Ambion) according to the manufacturer's instruction. First-strand cDNA was synthesized with 2 *μ*g of total RNA as a template using RevertAid First-Strand cDNA Synthesis Kit (Applied Biosystems). Briefly, 1 *μ*l oligo(dT)18 and 2 *μ*g total RNA from each sample were added to 9 *μ*l nuclease-free water, kept at 70°C for 5 min and then rapidly cooled. After that, the reaction system was successively added with 4 *μ*l 5× Mg^2+^ buffer, 2 *μ*l 10 mM dNTPs, 1 *μ*l RNA inhibitor, and 1 *μ*l reverse transcriptase. The condition of reverse transcription was set as follows: 25°C incubation for 5 min and 42°C for 60 min, followed by 85°C for 5 min so as to inactivate reverse transcriptase.

Quantitative analysis of iNOS and COX-2 mRNA expression was conducted with the ABI Step One Plus Real-time PCR System (Applied Biosystems), and FastStart Universal SYBR Green Master (Rox) (Roche) was utilized to detect PCR products. The specific sequences of the primers in the study were listed in Table 1. For PCR amplification, a volume of 25 *μ*l reaction mixture, which consisted of 2.0 *μ*l primer mix (containing 7.5 mM reverse and forward primers), 12.5 *μ*l SYBR Green Universal Master mix, 8 *μ*l nuclease-free water, and 2.5 *μ*l cDNA template, was subjected to predenaturization on the condition of 95°C for 10 min, followed by 40 cycles of denaturization at 95°C for 15 s and annealing at 60°C for 60 s. The melting curves were monitored at the end of the cycles. Each reaction was performed in triplicate. Glyceraldehyde-3-phosphate dehydrogenase (GAPDH) was used as the endogenous reference to normalize the expression levels of the target genes. The RT-PCR data were analyzed as 2^−△△Ct^ to determine the relative iNOS and COX-2 mRNA expression levels.

#### 2.7.4. Western Blot Analysis

Western blot analysis was performed to evaluate the effect of PAO on COX-2, iNOS, and NF-*κ*B signaling pathways according to the manufacturer's instructions. Briefly, the total protein, cytosol protein, and nuclear protein were extracted from paw tissues with a Nuclear-Cytosol Extraction Kit (Cell Signaling Technology, USA, resp.). The protein concentrations were measured with a BCA protein assay kit (Beyotime, China) to equalize protein extracts in each group. After that, 50 mg of protein extracts from each group was separated on 10% SDS-PAGE gel and electro-blotted onto the PVDF membrane. The PVDF membrane was then incubated with blocking solution (5% skim milk) at room temperature for 1 h so as to seal the unspecific binding sites. Subsequently, the membrane was incubated overnight with primary antibodies (Abcam Biochemical Co., Cambridge, UK) against COX-2, iNOS, p-IKK*β*, IKK*β*, p-I*κ*B*α*, I*κ*B*α*, p50, and p65, followed by incubation with secondary antibodies for 2 h at room temperature. Blots were developed by enhanced chemiluminescence detection (Amersham International plc., Buckinghamshire, UK), and the density of blots was quantified with Bio-Rad Quantity One Software. H3 and *β*-actin served as loading controls.

#### 2.7.5. Statistical Analysis

Statistical analysis was performed by Statistical Product and Service Solutions (SPSS) software (version 20.0). Data were subjected to one-way analysis of variance (ANOVA), followed by LSD's test. All data were expressed as means ± SEM. Values of *p* < 0.05 were considered statistically significant.

## 3. Results

### 3.1. GC-MS Analysis of Fresh and Stale Patchouli Oil

**Figure 1:**
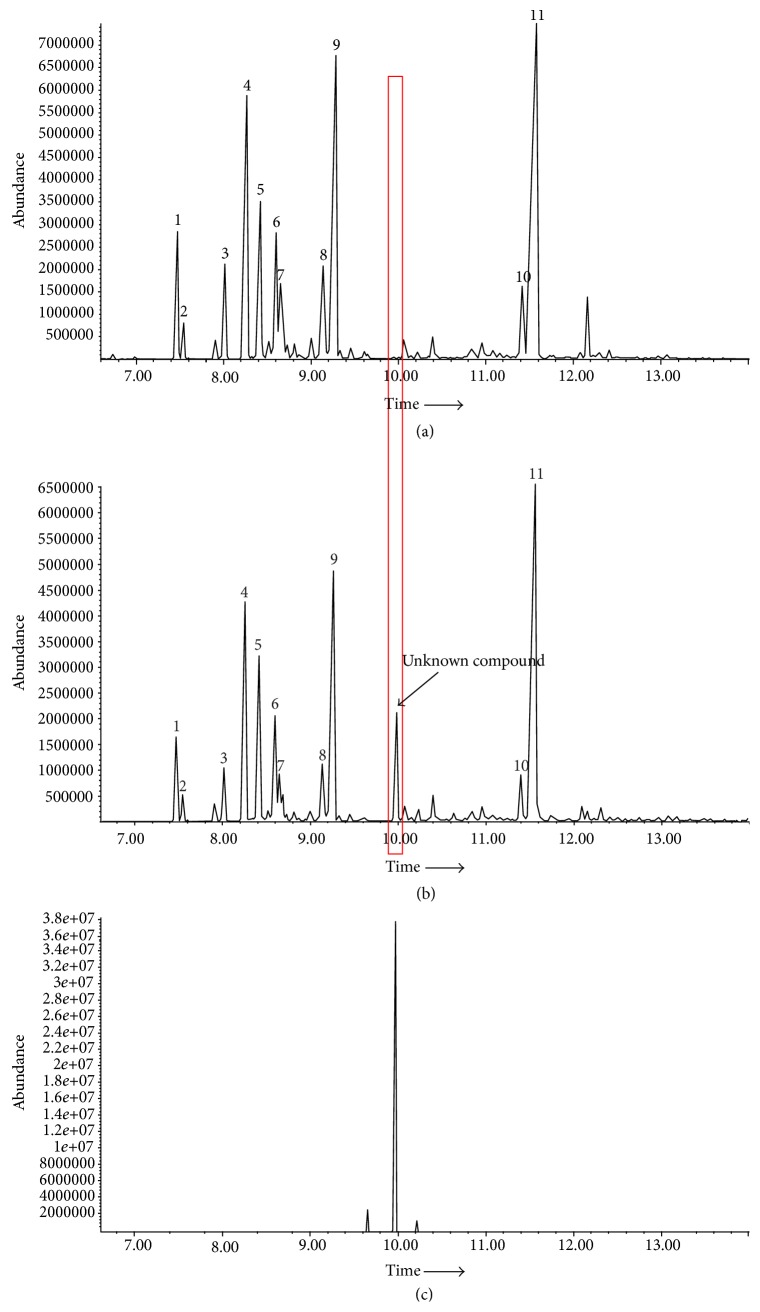
GC-MS analysis. (a) GC-MS analysis of fresh patchouli oil with one-year storage time. (b) GC-MS analysis of stale patchouli oil with ten-year storage time. Numbers indicated on peaks corresponded to those in Table 2. (c) GC-MS analysis of the unknown compound isolated from stale patchouli oil with ten-year storage time.

The total ion GC-MS chromatograms of fresh and stale patchouli oil were shown in Figures 1(a) and 1(b), respectively. Eleven compounds with relative content above 1% were identified in both fresh and stale patchouli oils. The retention indices and relative proportion of these eleven compounds were listed in Table 2. Furthermore, in the GC-MS spectrum of stale patchouli oil, an unknown compound with a content of 5.344% was observed at 9.996 min. However, no peak was found in the GC-MS spectrum of fresh patchouli oil during the corresponding retention time. After purification by fractional distillation, GC-MS analysis revealed that the purity of this unknown compound was over 95% (Figure 1(c)).

### 3.2. Identification of the Unknown Compound

**Figure 2:**
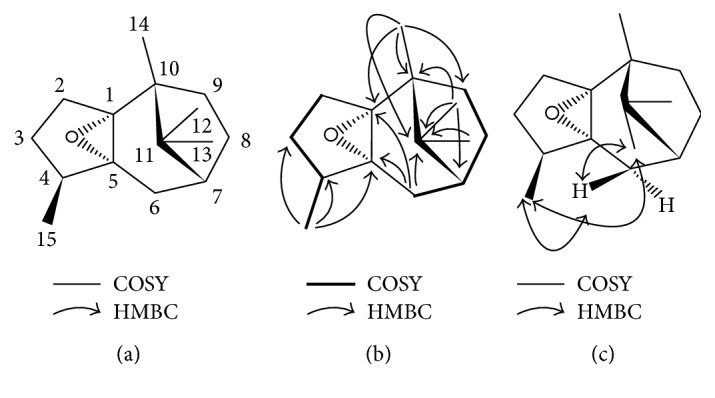
Identification of patchoulene epoxide (PAO). The chemical structure of PAO (a). Key ^1^H-^1^H COSY and HMBC correlations of PAO (b). Key NOESY correlations of PAO (c).

The unknown compound was isolated as a colorless transparent liquid. Its molecular formula was determined as C_15_H_24_O by HREIMS at *m/z* 220.1822. ^1^H-NMR (CD_3_OD, 400 MHz): *δ*_H_ 1.75 (1H, m, H-2a), *δ*_H_ 1.87 (1H, m, H-2b), *δ*_H_ 1.18 (1H, m, H-3a), *δ*_H_ 1.65 (1H, m, H-3b), *δ*_H_ 2.17 (1H, overlapped, H-4), *δ*_H_ 1.59 (1H, d, *J* = 14.8 Hz, H-6a), *δ*_H_ 2.17 (1H, overlapped, H-6b), *δ*_H_ 1.65 (1H, m, H-7), *δ*_H_ 1.32 (1H, m, H-8a), *δ*_H_ 1.81 (1H, m, H-8b), *δ*_H_ 1.40 (1H, m, H-9a), *δ*_H_ 1.98 (1H, ddd, *J* = 12.0, 9.3, and 3.1 Hz, H-9b), *δ*_H_ 0.87 (3H, s, H-12), *δ*_H_ 0.95 (3H, s, H-13), *δ*_H_ 1.06 (3H, s, H-14), and *δ*_H_ 1.03 (3H, d, *J* = 7.6 Hz, H-15). ^13^C-NMR (CD_3_OD, 100 MHz): *δ*_C_ 77.4 (C-1), *δ*_C_ 29.0 (C-2), *δ*_C_ 29.8 (C-3), *δ*_C_ 38.2 (C-4), *δ*_C_ 68.9 (C-5), *δ*_C_ 29.0 (C-6), *δ*_C_ 44.8 (C-7), *δ*_C_ 29.9 (C-8), *δ*_C_ 36.7 (C-9), *δ*_C_ 45.4 (C-10), *δ*_C_ 45.9 (C-11), *δ*_C_ 24.4 (C-12), *δ*_C_ 19.0 (C-13), *δ*_C_ 16.7 (C-14), and *δ*_C_ 17.6 (C-15). Consequently, this unknown compound was identified as patchoulene epoxide (PAO, Figure 2(a)). This was further confirmed by the key HMBC (Figure 2(b)) and NOESY (Figure 2(c)) correlations.

### 3.3. Acute Toxicity

Neither lethality nor abnormal behavioral indications were identified for mice with different doses of PAO during the acute toxicity test, which revealed that the LD_50_ value of PAO in mice was estimated to be above 10000 mg/kg. Therefore, there was a relatively wide margin of safety for PAO.

### 3.4. Effect of PAO on Xylene-Induced Ear Edema in Mice

**Figure 3:**
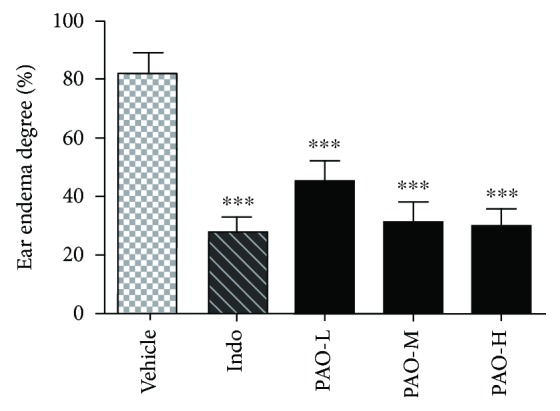
Anti-inflammatory effect of PAO on xylene-induced ear edema in mice. Indo: indomethacin (10 mg/kg); PAO-L: low dose of PAO (10 mg/kg); PAO-M: medium dose of PAO (20 mg/kg); PAO-H: high dose of PAO (40 mg/kg). Ear edema degree (%) was represented as the ratio of the difference in weight between the right and left ear of the same animal. Data were expressed as means ± SEM. (*n* = 10), and ^∗∗∗^*p* < 0.001 versus the vehicle group.

As shown in Figure 3, compared to the vehicle group, the ear edema degree was significantly lowered for mice with PAO and indomethacin pretreatment. The inhibitory rates of PAO were dose-dependently increased to 45.06% (*p* < 0.001), 61.75% (*p* < 0.001), and 63.62% (*p* < 0.001), respectively, while indomethacin diminished ear edema to 67.05% (*p* < 0.001).

### 3.5. Effect of PAO on Acetic Acid-Induced Vascular Permeability in Mice

**Figure 4:**
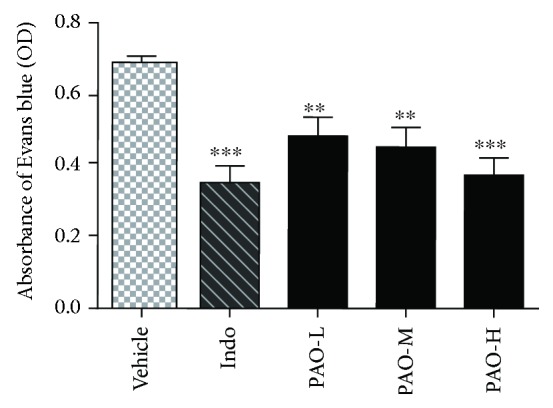
Anti-inflammatory effect of PAO on acetic acid-induced vascular permeability in mice. Indo: indomethacin (10 mg/kg); PAO-L: low dose of PAO (10 mg/kg); PAO-M: medium dose of PAO (20 mg/kg); PAO-H: high dose of PAO (40 mg/kg). The peritoneal vascular permeability was represented by the amount of Evans blue leaked into the abdominal cavity and measured by the absorbance of the supernatant at 590 nm. Data were expressed as means ± SEM. (*n* = 10), ^∗∗^*p* < 0.01, and ^∗∗∗^*p* < 0.001 versus the vehicle group.

As shown in Figure 4, PAO-L, PAO-M, and PAO-H caused a remarkable reduction in the acetic acid-induced extravasation of Evans blue dye when compared to the vehicle group, with inhibitory rates of 30.11% (*p* < 0.01), 34.56% (*p* < 0.01), and 45.87% (*p* < 0.001), respectively. Indomethacin also significantly decreased the dye leakage to 49.02% (*p* < 0.001).

### 3.6. Effect of PAO on Carrageenan-Induced Paw Edema in Mice

**Figure 5:**
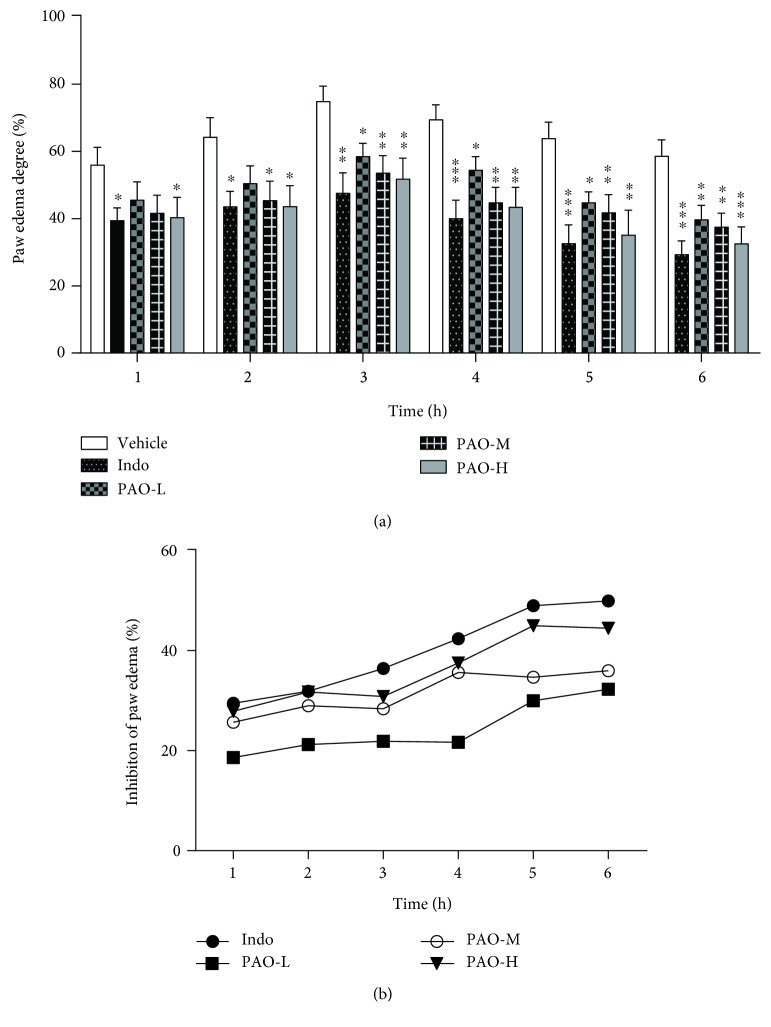
Anti-inflammatory effect of PAO on carrageenan-induced paw edema in mice. Indo: indomethacin (10 mg/kg); PAO-L: low dose of PAO (10 mg/kg); PAO-M: medium dose of PAO (20 mg/kg); PAO-H: high dose of PAO (40 mg/kg). (a) Paw edema degree was represented as the ratio of the paw volume variation between basal volume (0 h) and different time interval (1, 2, 3, 4, 5, and 6 h) volumes after carrageenan treatment. (b) Inhibition of paw edema (%) was represented as the paw volume difference between the vehicle group and the administration groups. Data were expressed as means ± SEM. (*n* = 10), ^∗^*p* < 0.05, ^∗∗^*p* < 0.01, and ^∗∗∗^*p* < 0.001 versus the vehicle group.

Intraplantar injection of carrageenan in mice led to a remarkable growth of paw volume in a time-dependent manner, and this increase was maximal at 3 h after carrageenan treatment (Figure 5(a)). The administration with PAO and indomethacin significantly inhibited the carrageenan-induced paw edema in all phases of the experiment when compared to that with the vehicle group (Figure 5(b)). Paw edema was suppressed by PAO-L and PAO-M, with inhibitory rates of 18.60–32.29% and 25.86–35.97%, respectively, during 1–6 h after carrageenan treatment. PAO-H and indomethacin generated a greater inhibition of edema development by 27.87–44.45% and 29.48–49.93%, respectively.

#### 3.6.1. Effect of PAO on the Levels of TNF-*α*, IL-1*β*, IL-6, IL-4, and IL-10

**Figure 6:**
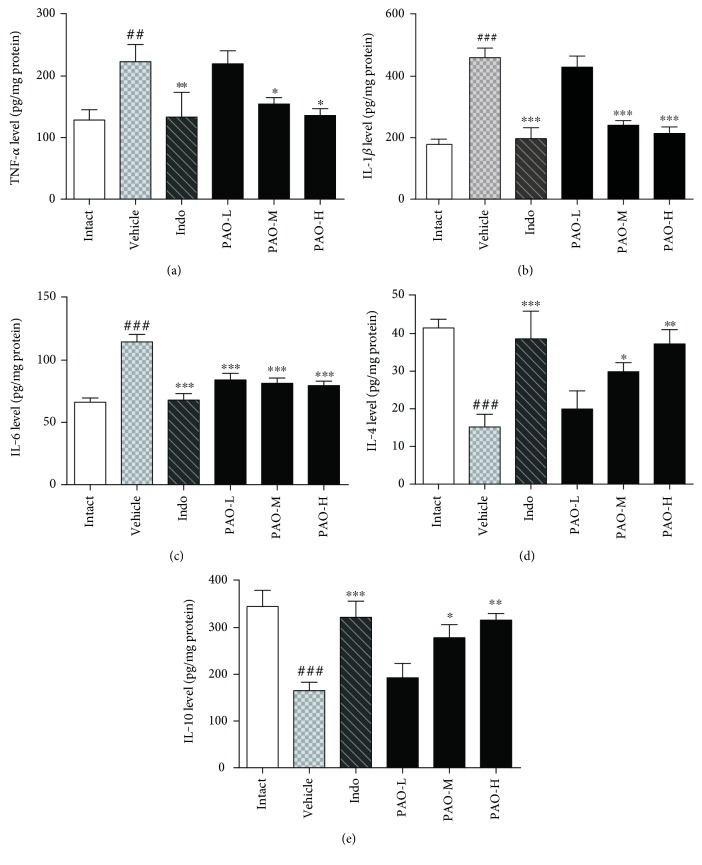
Effect of PAO on the levels of TNF-*α* (a), IL-1*β* (b), IL-6 (c), IL-4 (d), and IL-10 (e) in carrageenan-induced mouse paw. Indo: indomethacin (10 mg/kg); PAO-L: low dose of PAO (10 mg/kg); PAO-M: medium dose of PAO (20 mg/kg); PAO-H: high dose of PAO (40 mg/kg). Data were expressed as means ± SEM. (*n* = 10), ^##^*p* < 0.01, ^###^*p* < 0.001 versus the intact group. ^∗^*p* < 0.05, ^∗∗^*p* < 0.01, and ^∗∗∗^*p* < 0.001 versus the vehicle group.

Further investigation into the effects of PAO on the productions of pro- and anti-inflammatory cytokines was carried out. As shown in Figure 6(), the levels of proinflammatory cytokines including TNF-*α* (Figure 6(a)), IL-1*β* (Figure 6()), and IL-6 (Figure 6(c)) were significantly (*p* < 0.01) increased in the vehicle group to 1.74-, 2.59-, and 1.73-fold, respectively, when compared to the intact group. However, pretreatment with PAO-M, PAO-H, and indomethacin significantly (*p* < 0.05) reduced the levels of TNF-*α*, IL-1*β*, and IL-6. Contrarily, the levels of anti-inflammatory cytokines including IL-4 (Figure 6(d)) and IL-10 (Figure 6(e)) in the vehicle group were significantly (*p* < 0.001) decreased in the vehicle group to 2.73- and 2.09-fold, respectively, when compared to the intact group, while pretreatment with PAO-M, PAO-H and indomethacin can significantly (*p* < 0.05) reserve this trend. However, there were no obvious differences between the PAO-L group and the vehicle group on the levels of proinflammatory cytokines (TNF-*α* and IL-1*β*) and anti-inflammatory cytokines (IL-4 and IL-10).

#### 3.6.2. Effect of PAO on the Levels of PGE_2_ and NO

**Figure 7:**
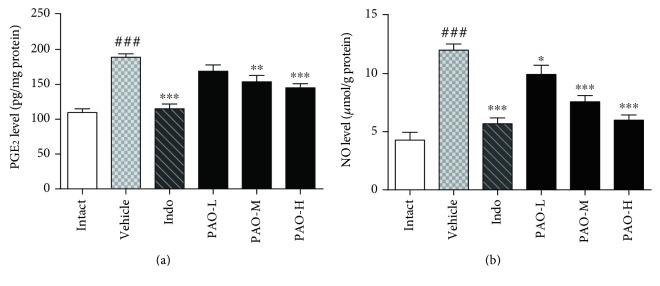
Effect of PAO on the levels of PGE_2_ (a) and NO (b) in carrageenan-induced mouse paw. Indo: indomethacin (10 mg/kg); PAO-L: low dose of PAO (10 mg/kg); PAO-M: medium dose of PAO (20 mg/kg); PAO-H: high dose of PAO (40 mg/kg). Data were expressed as means ± SEM. (*n* = 10), ^###^*p* < 0.001 versus the intact group. ^∗^*p* < 0.05, ^∗∗^*p* < 0.01, and ^∗∗∗^*p* < 0.001 versus the vehicle group.

As shown in Figure 7(a), compared with the intact group, the PGE_2_ level was increased markedly (*p* < 0.001) in the vehicle group. However, PAO-M and PAO-H significantly decreased the PGE_2_ level, with inhibitory rates of 17.78% (*p* < 0.01) and 22.27% (*p* < 0.001), respectively. Although the PGE_2_ level in the PAO-L group was also decreased to 9.62% when compared with that in the vehicle group, the difference was not statistically significant (*p* > 0.05). As shown in Figure 7(b), NO production was also significantly (*p* < 0.001) increased in the vehicle group when compared with the intact group. By contrast, PAO significantly suppressed the NO production in a dose-dependent manner with inhibitory rates of 17.34% (*p* < 0.05), 36.89% (*p* < 0.001), and 50.11% (*p* < 0.001), respectively. Indomethacin significantly suppressed the releases of PGE_2_ and NO, with inhibitory rates of 38.44% (*p* < 0.001) and 52.76% (*p* < 0.001), respectively.

#### 3.6.3. Effect of PAO on Protein and mRNA Expression of COX-2 and iNOS

**Figure 8:**
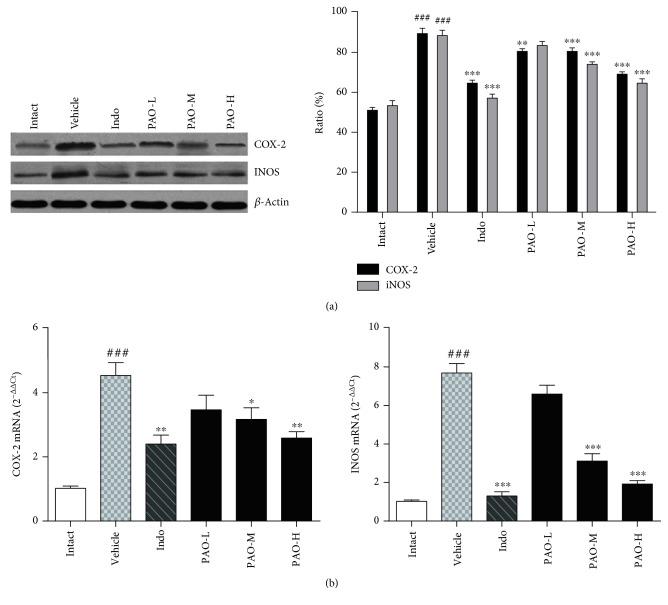
Effect of PAO on the protein and mRNA expressions of COX-2 and iNOS in carrageenan-induced mouse paw. Indo: indomethacin (10 mg/kg); PAO-L: low dose of PAO (10 mg/kg); PAO-M: medium dose of PAO (20 mg/kg); PAO-H: high dose of PAO (40 mg/kg). (a) Protein expression of COX-2 and iNOS was measured by Western blot analysis. *β*-Actin was used as a loading control. (b) Effect of PAO on COX-2 and iNOS mRNA expression. Total RNA was isolated and analyzed for mRNA expression via RT-PCR. Data were expressed as means ± SEM. (*n* = 3), ^###^*p* < 0.001 versus the intact group. ^∗^*p* < 0.05, ^∗∗^*p* < 0.01, and ^∗∗∗^*p* < 0.001 versus the vehicle group.

Western blot analysis and RT-PCR determination of COX-2 and iNOS were conducted. As shown in Figure 8(a), carrageenan treatment significantly (*p* < 0.001) upregulated the protein levels of COX-2 and iNOS when compared with the intact group, while PAO-M, PAO-H, and indomethacin pretreatment remarkably (*p* < 0.001) reversed this tendency. As shown in Figure 8(b), induction of inflammation by carrageenan dramatically (*p* < 0.001) increased COX-2 and iNOS mRNA expression as compared to that by the intact group. However, this effect was significantly (*p* < 0.01) suppressed by PAO-M, PAO-H, and indomethacin. Although PAO-L could also decrease the transcriptional levels of COX-2 and iNOS when compared with the intact group, the differences were not statistically significant (*p* > 0.05).

#### 3.6.4. Effect of PAO on the Expressions of NF-*κ*B Pathway-Related Proteins in Mouse Paw

**Figure 9:**
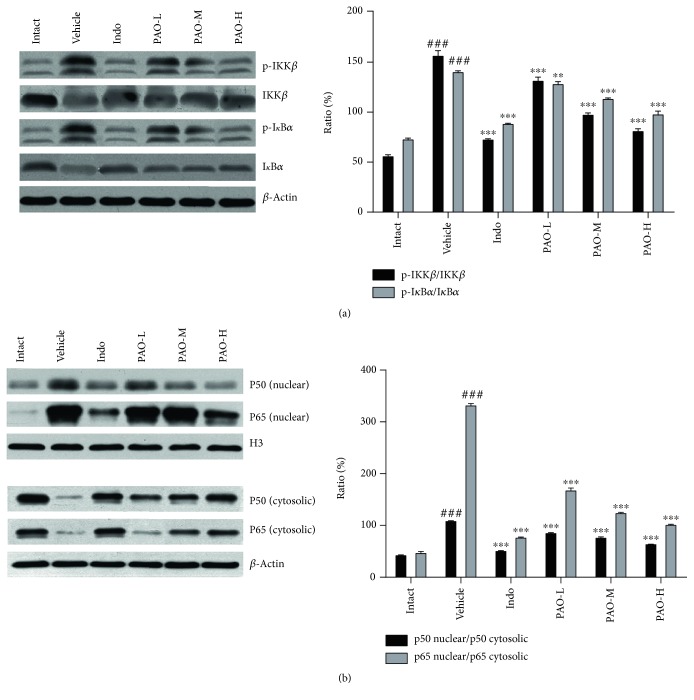
Effect of PAO on the expressions of NF-*κ*B pathway-related proteins in carrageenan-induced mouse paw. Indo: indomethacin (10 mg/kg); PAO-L: low dose of PAO (10 mg/kg); PAO-M: medium dose of PAO (20 mg/kg); PAO-H: high dose of PAO (40 mg/kg). Protein expression of p-IKK*β*, IKK*β*, p-I*κ*B*α*, I*κ*B*α*, and NF-*κ*B (p50 and p65) was measured by Western blot analysis. *β*-Actin and H3 were used as loading controls. Data were expressed as means ± SEM. (*n* = 3), ^###^*p* < 0.001 versus the intact group. ^∗∗^*p* < 0.01, and ^∗∗∗^*p* < 0.001 versus the vehicle group.

NF-*κ*B is a central transcription factor in the inflammatory cascade. To further expound whether the anti-inflammatory mechanism of PAO was associated with the inhibition of NF-*κ*B activation, the expression of NF-*κ*B pathway-related proteins was examined by Western blot. As shown in Figure 9(a), carrageenan treatment induced a striking (*p* < 0.001) increase in the phosphorylation patterns of IKK*β* and I*κ*B*α*, along with the decrease in the levels of IKK*β* and I*κ*B*α* when compared to the intact group. However, indomethacin and PAO pretreatments significantly (*p* < 0.01) decreased the ratio of p-IKK*β*/IKK*β* and p-I*κ*B*α*/I*κ*B*α* in a dose-dependent manner. Furthermore, the nuclear translocation of p50 and p65 was also measured. As shown in Figure 9(b), carrageenan treatment remarkably (*p* < 0.001) increased the translocation of p50 and p65 from the cytosol to the nucleus compared with the intact group. However, indomethacin and PAO significantly (*p* < 0.001) inhibited this translocation in a dose-dependent manner.

## 4. Discussion

By comparing the GC-MS spectrums of patchouli oil with different storage time, this study for the first time discovered an unknown compound only found in patchouli oil with ten-year storage time. The isolation of this compound was achieved by fractional distillation, and it was identified as PAO by 1D NMR, 2D NMR, and MS. It was reasonable to hypothesize that PAO might be an autoxidation production of *β*-patchoulene (PAE), since PAO could be directly synthesized from PAE [15, 16], which shared similar chemical structure with PAO. Previous researches on PAO have mainly concentrated on its chemosynthesis [16, 17]. However, its pharmacological effects still remain unexplored. In the present study, three murine experimental models were employed for the first time to demonstrate whether PAO had an anti-inflammatory effect as PAE.

Acute inflammation is a complex process, characterized by hemodynamic changes, enhancing vascular permeability and leukocyte infiltration [18]. To evaluate the potential effect of PAO on acute inflammation, the xylene-induced ear edema test was firstly conducted. Xylene is able to irritate the mouse ear instantly and induce fluid accumulation and neurogenic edema. Meanwhile, in response to the stimulation of xylene, many inflammatory mediators, including histamine, kinin, and fibrinolysin, will be released [19]. In this study, PAO pretreatment was observed to exhibit a significant dose-related inhibition on xylene-induced ear edema, suggesting that PAO might exert anti-inflammatory activity in the acute inflammation phase.

The acetic acid-induced peritoneal vascular permeability test is another well-recognized experimental model used for the evaluation of the anti-inflammatory effect of the candidates. Intraperitoneal injection of acetic acid into mice can result in peripheral endothelial cell shrinkage and dispersion. Afterwards, the permeability of the basement membrane will be correspondingly enhanced and allow many plasma proteins and fluid to pass into the injured tissues freely [20]. In this study, PAO remarkably reduced the enhancement of vascular permeability induced by acetic acid, which indicated that PAO might be able to reduce the vascular permeability and prevent intravascular substances from exudation.

The carrageenan-induced paw edema test is considered to be a sensitive acute inflammation model, which is generally used to explore the underlying mechanism of natural compounds. This model reveals edematization caused by the accumulation of leukocyte and the extravasation of fluid and proteins during early stages of acute inflammation [21]. Carrageenan-induced paw edema can be divided into two phases by within 120 min after carrageenan injection. The anterior phase involves the release of some chemical mediators including serotonin, histamine, and bradykinin, which mediate edema formation. The posterior phase is associated with the liberation of prostaglandins, cyclooxygenase, and other proinflammatory cytokines [22], which aggravates the inflammatory response. In this assay, PAO exerted potent inhibition of paw edema induced by carrageenan, which might be related to the decrease in fluid extravasation and mediator release. Based on these results above, it was valuable to further explore the anti-inflammatory mechanism of PAO.

TNF-*α*, IL-1*β*, and IL-6 are known as the most representative proinflammatory cytokines. Specifically, TNF-*α* is able to initiate other inflammatory mediators and thus augments the responses to inflammatory stimuli [23]. IL-1*β* has profound effects on inflammation and immunity and is responsible for cartilage destruction and bone resorption [24]. IL-6 contributes to inflammatory reaction by amplifying the recruitment of leukocytes and neutrophils, thus implicating a variety of inflammatory disorders [25]. According to our results, PAO could significantly decrease the levels of TNF-*α*, IL-1*β*, and IL-6. In addition, PAO was also capable of elevating the levels of anti-inflammatory cytokines including IL-4 and IL-10, which could reduce the productions of TNF-*α* and IL-6 and ultimately terminate inflammatory responses [26]. These results indicated that PAO might exert an anti-inflammatory effect, at least in part, via decreasing the levels of proinflammatory cytokines and increasing the releases of anti-inflammatory cytokines.

NO and PGE_2_ are commonly recognized as inflammatory indices, which can be regulated by TNF-*α* and IL-1*β* [27]. NO, generated by iNOS from L-arginine, is responsible for cell injury by producing reactive radicals like peroxynitrite. Our results revealed that PAO significantly suppressed NO release and downregulated iNOS expression in paw tissues. Furthermore, Mansouri et al. [23] revealed that NO also involved in catalyzing of COX-2 biosynthesis. The underlying mechanism possibly relates to lipid peroxidation initiated by peroxynitrite, which accounts for arachidonic acid liberation and COX-2 pathway activation [28]. PGE_2_, generated by COX-2 from arachidonic acid, is responsible for increasing vascular permeability and hemangiectasis. Besides, PGE_2_ is also associated with fluid leakage and protein extravasation [29]. Consistent with NO, PGE_2_ production and COX-2 expression were also significantly inhibited by PAO. Results above indicated that the anti-inflammatory effect of PAO might be associated with its inhibitory effect on NO and PGE_2_ production via blocking the iNOS and COX-2 transcriptional process.

Multiple reports have demonstrated that NF-*κ*B plays a pivotal role in inflammation acceleration by increasing various effector molecules such as TNF-*α*, IL-1*β*, iNOS, and COX-2 [30]. NF-*κ*B mainly comprises two subunits: p50 and p65. Under unstimulated conditions, both subunits exist primarily as cytosolic homo- or heterodimers complexed with inhibitory-*κ*B (I-*κ*B) protein [31]. In the canonical pathway of NF-*κ*B activation, IKK*β* is responsible for I*κ*B*α* phosphorylation [32]. With p-I*κ*B*α* being ubiquitinated and degraded by the proteasome, p50 and p65 are free to translocate from the cytosol to the nucleus and become the active forms [33], which can regulate the gene transcription of many inflammatory mediators [34]. Therefore, by inhibiting NF-*κ*B activation, TNF-*α*, IL-1*β*, iNOS, and COX-2 would also be suppressed. In this study, PAO not only significantly decreased the ratio of p-IKK*β*/IKK*β* and p-I*κ*B*α*/I*κ*B*α* but also markedly suppressed the translocations of p50 and p65 from the cytosol to the nucleus significantly. Taken together, PAO might exert an anti-inflammatory effect, at least partially, by inhibiting NF-*κ*B activation via blocking the phosphorylation cascades of IKK*β* and I*κ*B*α* and suppressing the translocation of p65 and p50. These findings provided a pharmacological basis for the anti-inflammatory effect of PAO and suggested that PAO might be a potential candidate for the therapy of inflammatory disorders.

## 5. Conclusions

Results of this study provided ample evidence for the anti-inflammatory property of PAO. Three experimental models were employed to demonstrate that PAO had potent anti-inflammatory activity in vivo. PAO exerted anti-inflammatory activity not only by modulating the pro- and anti-inflammatory cytokines but also by inhibiting iNOS and COX-2 activation via blocking the NF-*κ*B signaling pathway. Based on these results, PAO may serve as a potentially useful therapeutic agent for the treatment of inflammatory diseases, and it is worthwhile to explore other possible pharmacological effects of PAO.

## Figures and Tables

**Table 1:** Primer sequences.

Targeted gene	Direction and sequence
COX-2	F: 5′- GAAGATTCCCTCCGGTGTTT-3′
	R: 5′-CCCTTCTCACTGGCTTATGTAG-3′
iNOS	F: 5′-GGAATCTTGGAGCGAGTTGT-3′
	R: 5′-CCTCTTGTCTTTCACCCAGTAG-3′
GADPH	F: 5′-AGGAGCGAGACCCCACTAACA-3′
	R: 5′-AGGGGGGCTAAGCAGTTGGT-3′

**Table 2:** Components of common peaks in fingerprint of patchouli oil.

Peak number	Fresh patchouli oil		Stale patchouli oil		Components
	Retention time	Relative content (%)	Retention time	Relative content (%)	
1	7.469	7.457	7.469	3.861	*β*-Patchoulene (PAE)
2	7.542	1.154	7.546	1.114	*β*-Elemene
3	8.013	3.429	8.017	2.481	Caryophyllene
4	8.266	13.303	8.257	11.796	*α*-Guaiene
5	8.420	6.628	8.416	8.623	Seychellene
6	8.600	5.019	8.596	5.312	*α*-Patchoulene
7	8.647	3.163	8.643	1.982	*δ*-Guaiene
8	9.139	4.343	9.135	3.259	Globulol
9	9.285	17.449	9.264	14.366	*α*-Bulnesene
10	11.417	3.096	11.392	2.490	Azulene
11	11.580	27.804	11.559	28.183	Patchouli alcohol
